# Analysis of changes in “mitral valve reserve” after coronary artery bypass grafts in patients with functional mitral regurgitation

**DOI:** 10.1186/s13019-022-01993-6

**Published:** 2022-10-05

**Authors:** Fabrizio Ceresa, Antonio Micari, Antonino Salvatore Rubino, Liborio Mammana, Vito Pipitone, Giampiero Vizzari, Francesco Costa, Francesco Patanè

**Affiliations:** 1 Vascular and Thoracic Department, Papardo Hospital, Stagno d’Alcontress Street, 98121 Messina, Italy; 2 Division of Cardiology, G. Martino University Hospital, Messina, Italy; 3 Division of Cardiology, Papardo Hospital, Messina, Italy

## Abstract

**Introduction:**

The treatment of moderate functionalmitral regurgitation (FMR) during coronary artery bypass grafting (CABG) is still debated. Our primary end point was to assess the improvement of “mitral valve reserve” (MVR) after CABG alone as a clinical demonstration of left ventricular (LV) recovery.

**Materials and methods:**

Between June 2019 and June 2021, we prospectively enrolled 104 consecutive patients undergoing CABG with moderate FMR. Inclusion criteria were inferior-posterior-lateral wall hypokinesia and revascularization of the circumflex or right coronary artery. MVR was calculated as the ratio between anterior and posterior leaflets’ straight length. All patients were followed for 1 year. The improvement of MVR has been considered as a reduction of the ratio between anterior and posterior leaflets straight length.

**Results:**

Compared to baseline, mean MVR was significantly reduced both at 6 (2.24 ± 0.95 vs. 1,91 ± 0.6; p = 0,047) and 12 months follow-up (2.24 ± 0.95 vs. 1,69 ± 0.49; p = 0,006). Left ventricular (LV) reverse remodeling, meant as improvement of LV ejection fraction and reduction of LV end-systolic volume index and mitral anulus diameter were evaluated at 6 months and 1 year. Mitral regurgitation grade were also significantly reduced at 6 months (p < .001).

**Conclusion:**

The benefits of myocardial revascularization in term of improvement of mitral regurgitation’s degree can be explained by the changes of MVR. The patients with FMR, who could have more advantages from CABG alone, should be the ones who have LVESVi just moderately increased.

## Introduction

Functional mitral regurgitation (IMR) is a clinical condition secondary to left ventricular pathological remodeling, occurring in about 50% of patients with chronic coronary artery disease, being of moderate degree in almost 10% of cases [1, 2].

In normal valves, mitral valve coaptation line is asymmetric, with a clear anterior dominance [3]. In chronic ischemic MR, the increase in tethering forces, combined with the reduction of closing forces, displace the mitral leaflets, and the coaptation line is further transferred posteriorly [3, 4]. Despite the alteration of normal anatomy, mitral regurgitation is not a common finding. Indeed, Gogoladze et al. suggested that an increase in the movement of the anterior mitral leaflet can compensate such displacement, and thus postulated the existence of an “anterior leaflet” or “mitral valve reserve” [5].

Although in the last 10 years moderate FMR was considered a clear indication for surgery in patients undergoing CABG, the most recent evidences questioned the utility to treat the mitral valve in case of less than severe insufficiency. Accordingly, current guidelines equalized the qualitative and semi-quantitative echocardiographic parameters for the estimation of severity grade, supporting mitral valve surgery only in case of severe insufficiency [6, 7]. On the other hand, the most appropriate treatment for moderate FMR in patients undergoing CABG is still debated.

In this study, we sought to investigate changes in mitral valve reserve in patients with moderate FMR undergoing isolated CABG.

## Materials and methods

### Study population and inclusion criteria

Between June 2019 and June 2021, data from all patients undergoing isolated CABG were prospectively enrolled in an institutional database.

**Fig. 1:**
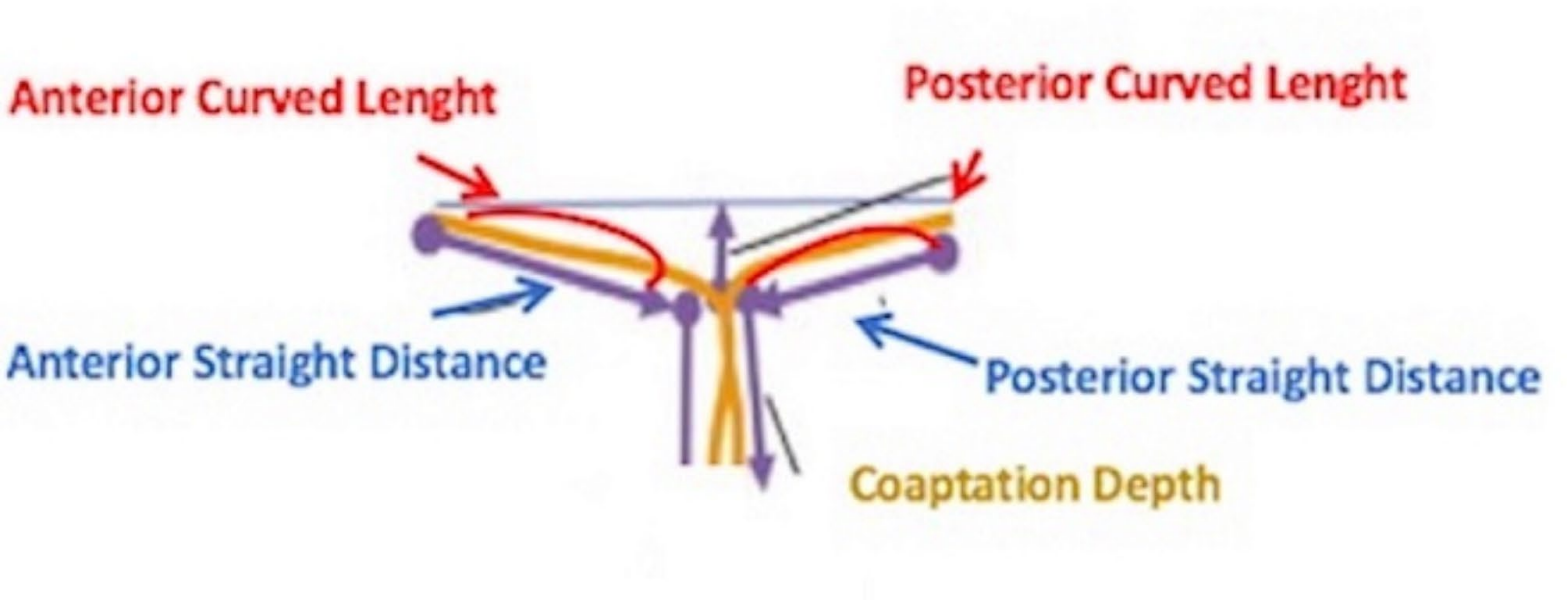
The schematic drawing of the mitral valve shows how the Anterior (AL) and Posterior Leaflet (PL) straight length, mitral anulus diameter (An) and coaptations Depth (CD) were usually measured from the apical four-chambers view at transthoracic echocardiography

All patients underwent preoperative transthoracic echocardiography. Intraoperative transesophageal echocardiography was always performed to confirm the absence of a mitral-valve structural abnormality. Mitral valve anterior and posterior leaflets’ straight length, the coaptation depth (CD) and the septo-lateral anulus diameter (distance from anterior to posterior anulus) were measured from the apical four chamber view. Mitral valve reserve was calculated as the ratio between the anterior and posterior leaflets’ straight length [8] (Fig. 1). The ejection fraction and left ventricular end-systolic volume index (LVESVi) were recorded as well. Pharmacological stress test was performed to confirm myocardial vitality in patients with borderline segmental wall motion.

For the specific aim of this study, the selection criteria were moderate functional mitral regurgitation, hypokinesia of the left ventricular inferior-posterior-lateral region and the ability to revascularize of one or more branches of circumflex and/or right coronary arteries. Mitral valve surgery was not performed, according to current guidelines [6, 7].

Patients with akinesia, reduced thickness, aneurysm, scar and absence of myocardial vitality at myocardial stress test of the of left ventricular inferior-posterior-lateral wall or severe ischemic mitral regurgitation were excluded.

The degree of mitral valve regurgitation was defined according to vena contracta width. The mechanism of mitral regurgitation was an asymmetric tethering of the posterior leaflets in all patients. A single imaging specialist evaluated all echocardiograms blinded to patients’ characteristics and type of interventions performed.

Among 531 patients included, 104 fulfilled the inclusion criteria and were part of the present analysis.

All patients were prospectively followed for 2 year. Mitral valve function and left ventricular remodeling were assessed at baseline, at 6 and 12 months postoperatively.

### Endpoints of the study

The primary endpoint was the improvement of mitral valve reserve, defined as the reduction of the ratio between the anterior and posterior leaflet length [8]. The secondary endpoints were the occurrence of left ventricular reverse remodeling, defined as a reduction of LVESVi, the improvement of ejection fraction (EF) and the assessment of the degree of mitral regurgitation.

### Statistical analysis

Continuous variables are expressed as means and standard deviation, whereas categorical variables are reported as counts and percentages. Change over time of mitral valve reserve, leaflet lengths, annular septo-lateral diameter, LVESVi, LVEF and degree of MR were assessed with repeated measure ANOVA and Friedman’s two was analysis of variance by ranks.

Data were analyzed with SPSS 24 (SPSS Inc, Chicago IL). Statistical significance was set at a p-value < 0.05.

## Results

Patients’ preoperative characteristics are summarized in Table 1. Mean number of grafts performed was 3,33 ± 0,91. Mean follow-up was 19,64 ± 8,5 months (min 4,43 months; max 30,6 months).

**Table 1:** Baseline characteristics of the patients *

Characteristic	CABG alone (N=104)
Age (y)	68,18 + 10,57
Male sex – no (%)	92 (88,4%)
Diabetes – no (%)	57 (54,8%)
Hypertension – no (%)	77 (74%)
COPD – no (%)	15 (14,4%)
Previous Myocardial infarction – no (%)	24 (23,1%)
Left ventricular end-systolic volume index – ml/m2	30,69 + 16,6
Left ventricular ejection fraction – %	44,0 + 11%
BSA- m2	1,9 + 0,2
Creatinine value – mg/dl	0,95 + 0,35
Atrial Fibrillation (AF) - %	8 (7,4%)
Mild Mitral Regurgitation Degree – (%)	65 (62,5%)
Moderate Mitral Regurgitation Degree – (%)	39 (37,5%)
Left Atrial Area (LAA) – cm^2^	24,1 + 6,7
Vena Contracta (VC) width – mm	3,6 + 1,9
LV diastolic diameter - mm	50,2 + 7,6
Mean PAP - mmHg	28 + 6,6

*CABG denotes coronary artery bypass grafting, COPD chronic obstructive pulmonary disease, BSA body surface area

All patients were successfully discharged from hospital. During follow-up, four patients died for non-cardiac-related cause. Accordingly, follow-up was 96.1% and 48% complete respectively at 1 years and 2 years.

When mitral valve was considered, posterior leaflet length significantly change overtime, whereas no significant changed in anterior leaftlet was observed (Table 2). Conversely, mitral annular septo-lateral distance reduced significantly at 6 months (37.6 ± 4.5 vs. 35.4 ± 3.9, p = .025), and remained stable at 1 year. Consequently, mitral valve reserve significantly improved both at 6 months and 1 years compared to baseline (2.24 ± 0.95 vs. 1,91 ± 0.6; p = 0,047 and 2.24 ± 0.95 vs. 1,69 ± 0.49; p = 0,006 respectively), as well as the coaptation depth.

**Table 2:** Clinical End Points

End Point	Baseline (N = 104)	6 months (N = 100)	1 year` (N = 100)	P Value
AL straight length - mm	23,15 + 2,8	22,9 + 3,3	22,5 + 3,5	p = 0,6
PL straight length*,**,*** – mm	11,23 + 2,5	12,85 + 3,5	14,03 + 3,5	p < .0001
Mitral valve reserve*,**,***	2,24 + 0,95	1,91 + 0,60	1,69 + 0,50	p < .0001
Mitral anulus – mm*,**	37,7 + 4,6	35,4 + 3,9	34,6 + 3,3	p = 0,008
Coaptation Depth, mm	3,6 + 1,5	4,6 + 1,2	4,4 + 1,2	P =.01
LVEF - %*,**,***	44,0 + 11	47,6 + 10,1	50,2 + 8,9	P < .0001
LVESV index – ml/m2	30,7 + 16,6	28,2 + 14,1	24,4 + 8,4	p = 0,069
LEDV index – ml/m^2^	63,6 + 6,2	57,82 + 5,2	54,78 +3,9	p= 0,094
no MR degree – %	0 (0%)	10 (10%)	24 (24%)	
Mild MR Degree - % -	65 (62,5%)	60 (60%)	76 (76%)	
Moderate MR degree -%_	39 (37,5%)	30 (30%)	0 (0%)	
VC width^*,**,***^ - mm	3,6 + 1,9	2,2 + 0,9	1,5 + 0,3	p < .0001

AL denotes anterior leaflet, PL posterior leaflet, LVEF left ventricular ejection fraction, LVESV index left ventricular end-systolic volume, VC Vena Contracta

*baseline vs. 6 months p< .05

** baseline vs. 12 months p< .05

***6 months vs. 12 months p< ,05

The rank-based assessment of mitral regurgitation’s degree at 6 months over showed a significant reduction compared to baseline (p < 0,001): particularly, at 6 months the mitral insufficiency was mild or no in the 70% of patients. At 1 year all followed patients had mild or no mitral valve regurgitation as well as at 2 years. At the admission, 23% of patients were in NYHA > 3. At 1 and 2 years all followed patients were in NYHA 1.

**Fig. 2:**
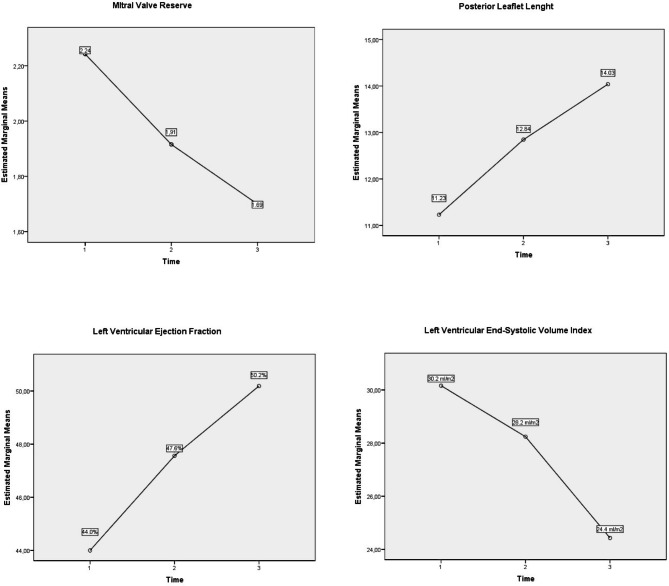
The time trend of the mean values of Mitral Valve Reserve, the Posterior Leaflet straight length, the LV ejection fraction and LV end-systolic volume index plotted on the graphs. The number 1,2 and 3 on the x-axis mean respectively baseline, 6 months and 1 years follow-up

Left ventricular reverse remodeling was evident at follow-up with a borderline reduction of LVESVi (p = 0,06 and significant increase of LVEF at 6 and 12 months (44.0 ± 11.1% vs. 47,56.±10,12, p = 0,018; 44.0 ± 11.1% vs. 50,19.± 8,87 p < 0,001, respectively baseline vs. 6 months and baseline vs. 1 year). (Fig. 2)

Afterwards, patients were divided in two groups, based on the baseline mitral regurgitation’s degree (Group 1: mild MR vs. Group 2: moderate MR). The baseline characteristics of two groups are summarized in the Table 3. Mean anulus diameter and the posterior leaflet straight length are significantly longer in the moderate MR group than in the mild MR group. In the moderate MR group, the CD is significant shorter than the mild MR group. Mitral valve reserve significant increase in both groups at 12 months compared to the baseline (Group 1: 2.06 ± 0,9 vs. 1,65 ± 0,4; p = .002: Group 2: 2.26 ± 0,4 vs. 1,87 ± 0,8; p < .05). In the moderate MR group, all followed patients have mild mitral regurgitation at 12 months.

**Table 3:** Baseline Characteristics

	**Group 1** **(Mild MR)** **N = 65**	**Group 2** **(Moderate MR)** **N = 39**	**P value**
Age (y)	67,1 + 9,2	70,1 + 12,6	0.40
Male sex – N(%)	60 (92,3)	36 (92,3)	0.62
Diabetes – N (%)	40 (61,5)	20 (51,3)	0.37
Hypertension – N (%)	44 (67,7)	36 (92,3)	0.08
COPD – N (%)	12 (18,5)	4 (10,2)	0.39
Left ventricular end-systolic volume index – ml/m2	28,58 + 12,9	36,59 + 24,1	0.34
Left ventricular ejection fraction – %	44,9 + 10,3	39,7 + 11,8	0.11
Atrial Fibrillation (AF) - %	4 (6,1)	4 (10,2)	0.54
AL straight length - mm	22,6 + 2,9	21 + 6,9	0.34
PL straight length – mm	11,9 + 2,5	9,3 + 3,5	0.008
Mitral valve reserve	2,26 + 0,4	2,06 + 0,9	0.27
Mitral anulus – mm	35,7 + 4,1	40,6 + 3	0.001
Coaptation Depth, mm	4,9 + 1,7	3,9 + 1,6	0.03

## Discussion

In the current study from a highly selected cohort of patients with moderate FMR, undergoing target vessel revascularization without concomitant valve repair, we observed an improvement of mitral valve reserve, reduction of valve insufficiency and improved LV performance at short-term follow-up.

Occurrence of ischemic mitral regurgitation depends on an imbalance between increased tethering forces and reduced closing forces, the latter including reduction in left ventricular (LV) contractility and altered systolic annular contraction. [9] The posterior papillary muscle is most often involved in ischemic cardiomyopathy because only one vessel, arising from circumflex or right coronary artery, supply to its vascularization. The abnormal function of the LV inferior-posterior-lateral region or the LV remodeling due to myocardial infarction cause a posterior-lateral and apical displacement of the posterior papillary muscle thereby increasing the tethering forces. [10, 11]

Moderate FMR is common in patients needing myocardial revascularization, ranging from 10 to 50% [12]. In the most recent years, the usefulness of addressing moderate functional regurgitation concomitantly to CABG has been questioned: many observational studies and a randomized clinical trial of Cardiothoracic Surgical Trials Network (CTSN) showed the benefits of mitral valve repair during CABG by lowering the incidence or recurrence of MR [13, 14], that came at the cost of an increasing rate of adverse neurologic events, longer Intensive Care Unit (ICU) stay and supraventricular arrhytmias [15–17].

Although a general agreement exists about the need to treat severe mitral valve insufficiency, the functional etiology of this peculiar entity, that is left ventricular dysfunction, may suggest to focus the surgical attention more on the recovery of myocardial perfusion and vitality rather than only restricting the annulus of a mitral valve with almost anatomical intact leaflets. Indeed, in the normal mitral valve, the leaflet’s contribution to the coaptation is asymmetric for the dominance of the anterior one as pointed out by Gogoladze G. et al. [5]. FMR is at least partially associated to leaflet restricted movement that led to the changes in mitral leaflet opening and closing cycle. The coaptation line is transferred posteriorly and mitral valve remains continent until the anterior leaflet is able to compensate the tethering of the posterior leaflet. This pathophysiological mechanism, that leads to the reduction of leaflets CDs and increasing of coaptation depth, suggests that an “anterior leaflet or mitral valve reserve” exists [3]. Several methods have been described to calculate mitral valve reserve. Mahmood F. and colleagues measured the anterior curved length and straight distance, posterior curved length and straight distance and the coaptation depth (CD) at transesophageal echocardiography, repeating the measurements three times for every mitral valve region [8]. Although very precise and useful for research purposes, this formula is too complex to be used in the daily clinical practice. Therefore, we have simplified it as a ratio between anterior and posterior straight length, measured from the apical four-chambers view at transthoracic echocardiography [10].

The reduction of “mitral valve reserve” observed in the present study supports the knowledge that functional insufficiency is primarily a ventricular disease, rather than a leaflet disease. It could be speculated that the significant reduction of the ratio between the anterior and posterior leaflet straight lengths might be explained by the anterior relocation of the coaptation line as a consequence of the recovery of left ventricular inferior-posterior-lateral contractility leading to the improvement of the posterior leaflet motion.

Although the study might be underpowered because of the small size of the sample, target myocardial revascularization allows achieving a positive reverse left ventricular remodeling, with reduced LVESVi and annular diameters, as well as an increase in LVEF, provided the evidence of underlying vital myocardium.

The observed reduction of mitral regurgitation grade, which has been already described after CABG [18–21], may thus be the consequence of the concurrent contribution of left ventricular reverse remodeling and the changes of mitral valve reserve, with stable results up to 2 years.

In our experience, we did not observe any worsening of mitral regurgitation during follow-up so that as the insufficiency was mild in the 70% of patients at 6 months. All followed patients had mild or no mitral regurgitation at 12 months and 2 years.

Comparing our results to similar surgical series, we observed a greater reduction of mitral regurgitation degree than in other studies. Indeed, Smith P.K and colleagues found that the 69% of patients had no or mild mitral valve regurgitation at 12 months in the group of CAGB alone as compared with 89% of patients in the group of CABG plus mitral valve repair. [12] Similar results were achieved by Kang D.H and colleagues, who didn’t find any difference in term of improvement of mitral degree regurgitation between the groups of patients with moderate mitral regurgitation undergoing a CABG alone or a combined-procedures (65% vs. 76% respectively; p = ns) [22–24].

It may be speculated that the more evident reduction of mitral regurgitation grade might depend on the lower left ventricular volumes at baseline recorded in our study rather than the result of the published trials. [13–17]

Reserve LV remodeling is well known to have been associated with an improved outcome, although, a recent randomized trial showed that there is no significant difference between patients undergoing CABG alone and combined treatment [15].

Moreover, the data published in the literature show that the recurrence of moderate or severe mitral valve regurgitation after CABG with mitral valve repair is roughly 30% at 2 year.

Currently, scientific evidence suggest that the addition of mitral valve repair in patients undergoing CABG with moderate FMR had no incremental effect on reverse LV remodeling and survival. [12]

### Limitations

The small sample size is the main limitation of our study, which might underpower our results. Accordingly, we tried to overcome such limitation with strict inclusion and exclusion criteria, in order to limit as much as possible the interferences of unaddressed confounders. On the other hand, the single center design guarantees uniformity of surgical technique, data collection and analysis.

## Conclusion

Our results show an improvement of the MVR that explain the significant reduction of the degree of mitral valve regurgitation.

In our experience, the patients with moderate FMR, who benefit more from isolated CABG, are those with only moderate increased LVESVi.

Further studies, specifically designed to address the pathophysiological changes of left ventricular remodeling also in terms of mitral valve reserve, are needed to identify an optimal LVESVi cut-off for treatment decisions in moderate ischemic mitral regurgitation.

## Data Availability

The data that support the findings of this study are not openly available because they are human data and are available from the corresponding author upon reasonable request.
